# Sexual risk and STI testing behaviour among Dutch female and male self-employed sex workers; a cross-sectional study using an Internet based survey

**DOI:** 10.1186/s12889-022-13582-2

**Published:** 2022-06-09

**Authors:** C. J. G. Kampman, C. M. M. Peters, F. D. H. Koedijk, T. S. Berkenbosch, J. L. A. Hautvast, C. J. P. A. Hoebe

**Affiliations:** 1 Public Health Service Twente, Postbus 1400, JM 7511 Enschede, The Netherlands; 2 Public Health Service South Limburg, Heerlen, The Netherlands; 3 Faculty of Health, Medicine and Life Sciences, Department of Social Medicine and Medical Microbiology, Maastricht University, Care and Public Health Research Institute, Maastricht, The Netherlands; 4 Department of Primary and Community Care, Radboud University Medical Centre, Nijmegen, The Netherlands

**Keywords:** Sex workers, Sexual health, STI, Sexually transmitted infections

## Abstract

**Background:**

Sex workers are men, women or transgender people who have sex in exchange for money or goods. Self-employed sex workers solicit clients independently from a third-party. Self-employed sex workers are at risk of acquiring sexually transmitted infections (STIs) through their work.

We performed a cross-sectional study, using an Internet survey conducted in 2019–2020 aiming to establish sexual risk behaviour and STI testing behaviour among female and male self-employed sex workers.

**Results:**

A total of 76 female self-employed sex workers (FSW) and 79 male self-employed sex workers (MSW) completed the survey. Both FSW and MSW more often had sex with partners of the opposite sex during work (65.8% FSW, 61.6% MSW) and in their private life (63.3% FSW; 64.5% MSW). During vaginal sex 35.7% of FSW and 29.6% of MSW did not always use a condom. Inconsistent condom use was observed in 35.7% of FSW and 29.6% of MSW during vaginal sex, 46.2% of FSW and 35.7% of MSW did not always use a condom during receptive anal sex. The majority of both FSW and MSW tested for STIs in the past year (67.1% FSW; 67.7% MSW) and 67.5% were aware of the possibility of low-threshold testing at an STI clinic. In the past year, 11.6% of FSW and 8.1% of MSW had an STI.

**Conclusion:**

The reported STI positivity rate among self-employed sex workers was not very high. However, STI prevention efforts remain important considering the low compliance with condom use during sex work. Moreover, not testing for STIs in the past year was substantial with one-third of both FSW and MSW and one-third of both FSW and MSW being unaware of the possibility of low-threshold testing at an STI clinic, warranting efforts to increase testing uptake in this population.

## Key messages

- Self-employed sex workers solicit clients independently of a third-party. This study included 76 female and 79 male self-employed sex workers.

- Inconsistent condom use occurred in 35.7% of FSW and, 29.6% of MSW with vaginal sex and, 46.2% of FSW and 35.7% of MSW with receptive anal sex.
- The majority of both FSW (67.1%) and MSW (67.7%) tested for STI in the past year, and 11.6% of FSW and 8.1% of MSW reported having had an STI.
- STI prevention efforts remain important, considering the low compliance to condom use and the large number of sexual contacts that might facilitate STI spread.

## Background

Sex workers are men, women or transgender men or women who receive income, employment, survival (e.g., food or shelter), and/or drugs in exchange for sexual services [1–3]. Sex workers are of public health importance because of their high occupational risk of acquiring sexually transmitted infections (STIs), including human immunodeficiency virus (HIV), and their risk for further spreading of these STIs through clients and private partners to the general population [1, 4].

Several studies have concluded that inconsistent condom use can contribute to the spread of STIs [5–7]. A systematic review and meta-analysis performed among female sex workers (FSW) globally reported 19.1% (95% CI: 1.7 to 36.4) engaged in unprotected vaginal sex and 46.4% (95% CI: 9.1 to 83.6) engaged in unprotected anal sex [8]. A Dutch study performed in three cities reported 81% of sex workers exhibited consistent condom use among female and male-to-female transgender sex workers [4]. Another Dutch study suggested that almost half of all sex workers had engaged in condomless anal sex with clients in the past 6 months [9].

Inconsistent condom use is one of the factors leading to high STI positivity rates among sex workers worldwide [10–12]. However, STI positivity rates are lower in Western countries than in other countries. A progress report by the World Health Organization showed that 11% of sex workers worldwide acquired HIV in 2020, while in the Netherlands the HIV prevalence among FSW was estimated to be 1.5%, and only 1.1% of male sex workers (MSW) tested positive for HIV in 2019 in the Netherlands [4, 12, 13]. Furthermore, another Dutch study showed that 20.3% of MSW having sex with men and 20.0% of transgender sex workers were known to be HIV-positive, compared with none of the MSW having sex with women [9]. The STI positivity rate among FSW remained relatively stable at approximately 9.5% in the Netherlands between 2006 and 2013 [14]. Other Dutch studies reported 29–40% STI positivity among MSW having sex with men, 26% STI positivity among transgender sex workers and 13% STI positivity among MSW having sex with women, compared with 9% among FSW [9, 15].

Sex workers can be employed by a third-party, such as an agency, manager, or gatekeeper (pimp), or they can be self-employed. Self-employed sex workers solicit clients independently from a third-party [2, 4, 16]. These self-employed sex workers are harder to reach for healthcare workers, because they are not organized or centralized such as sex workers who work in a formal indoor or outdoor setting, where they can be reached by outreach activities [2]. Furthermore, Dutch legislation only legalizes sex work with a permit, leaving limited work options without a permit for self-employed sex workers and forcing them to work in illegal circuits, which increases the risks of exploitation and unsafe work conditions [2, 17]. The STI clinics in the Netherlands offer free and anonymous STI testing for sex workers, but it is not known whether self-employed sex workers are reached by STI clinics. Consequently, this high-risk population is presumably hidden in sexual health care efforts.

To gain more insight into the group of female and male self-employed sex workers, we performed a cross-sectional study, using an Internet-based survey to assess and compare sexual risk behaviour and STI testing behaviour between female and male self-employed sex workers and to assess determinants of reported STI testing in the past year. The study outcomes can be used to gain insight into this hidden population, possibly at risk for STIs, and to inform public health professionals about STI prevention and tailoring sexual healthcare services for this population.

## Methods

### Study design, population and data collection

We performed a cross-sectional study, using an Internet survey conducted in the Netherlands from 2019 to 2020. The Internet survey consisted of questions on sociodemographic characteristics, sexual behaviour, and alcohol and/or drug use while working. The Internet survey was advertised throughout March 2019 with a banner on three national websites where sex workers advertise themselves. Additionally, the mobile phone numbers of sex workers advertising on the Internet, were used to send text messages with a link to our survey, or were made attentive to our survey during STI clinic visits and outreach activities.

The participants were asked to participate in the survey only once. Participants who met the definition of self-employed sex workers, were aged 18 years or older and performed sex work in the past 6 months were included in the analyses. In this study, we define FSW, MSW, and transgender sex workers (TSW) as self-employed sex workers if they had engaged in sex in exchange for money or goods and solicited clients independently of a third-party.

The incentive to participate in the study was the allotment of 50 Euros at the end of the study period. The survey software program SurveyMonkey (San Mateo, California, USA) was used to embed the questions and provide data for analysis.

### Variables

We assessed the demographic variables of country of birth and the reported highest level of education, relationship status, and gender of the sex partners in private life and in work setting. Educational level was defined as follows: low educational level was pre-primary education, primary education, or first stage of basic education; intermediate educational level was lower secondary education, or second stage of basic education; and high educational level was upper secondary education or tertiary education.

Furthermore, we assessed work-related variables, such as; work years, work frequency, reasons for self-employed sex work, other job besides sex work, main jobs, and work location. In addition, the following STI risk behaviour variables were assessed; types of sex during work and inconsistent condom use during work. When participants selected “never” or “not always” using a condom during work, this was considered to be inconsistent condom use. Receiving oral sex and insertive anal sex were assessed for MSW only, unprotected sex by client demand, asking more money for unprotected sex, asking more money for special request, alcohol or drug use during work, more often unprotected sex with alcohol or drugs and group sex were STI risk behaviour variables. Finally, we assessed STI testing behaviour variables: self-reported STI testing in the past year, STI test location, reasons for STI testing, and having had an STI in the past year. Having had an STI in the past year was based on self-reporting and chlamydia, gonorrhoea, syphilis, HIV, hepatitis B, genital warts, herpes, trichomonas, and scabies were considered STIs.

### Data analysis

Descriptive analyses were performed for all variables. The χ2 test was used to test for differences in proportions between male and female sex workers, as well as factors for being tested for STI in the past year. A *p*-value of < 0.05 was considered to be statistically significant in both analyses. Analyses were conducted using SPSS for Windows (version 26.0; IBM Inc., Somers, New York, USA).

## Results

### Study population

A total of 316 individuals started the survey, 153 of whom were excluded based on an exclusion criterion (i.e., being younger than 18 years, not being self-employed, and/or did not perform sex work in the past 6 months), and 163 met the inclusion criteria and participated in the survey (51.6%). There were 79 FSW (48.5%), 76 MSW (46.6%) and 8 TSWs (4.9%). In Table 1, we only display the FSW and MSW, as the TSW were not included owing to low numbers.

**Table 1:** Self-reported demographic and behavioural characteristics of self-employed female and male sex workers in the Netherlands (2019–2020)

	**Female sex workers** n(%)^a^	**Male sex workers** n(%)^a^	**Total** n(%)^a^	***p*-value**
**Demographics**				
**Country of birth**				**0.043**
The Netherlands	52 (65.8)	61 (80.2)	113 (72.9)	
Other country	27 (34.2)	15 (19.7)	23 (14.8)	
**Educational level**				0.154
Low educational level	14 (17.7)	6 (7.9)	20 (12.9)	
Intermediate educational level	37 (46.8)	36 (47.4)	73 (47.1)	
High educational level	28 (35.4)	34 (44.7)	62 (40.0)	
**Relationship status**				**0.021**
Relationship	36 (45.6)	21 (27.6)	57 (36.8)	
Single	43 (54.4)	55 (72.4)	98 (63.3)	
**Gender sex partners in private life**				0.979
Same sex	6 (7.6)	6 (7.9)	12 (7.7)	
Opposite sex	50 (63.3)	49 (64.5)	99 (63.9)	
Both opposite and same sex	23 (29.1)	21 (27.6)	44 (28.4)	
**Gender work sex partners**				0.085
Same sex	2 (2.7)	9 (12.3)	11 (7.5)	
Opposite sex	48 (65.8)	45 (61.6)	93 (63.7)	
Both opposite and same sex	23 (31.5)	19 (26.0)	42 (28.8)	
**Sex work**				
**Work years**				**0.027**
< 1 year	11 (16.4)	21 (36.8)	32 (25.8)	
1–5 year	33 (49.3)	24 (42.1)	57 (46.0)	
> 5 year	23 (34.3)	12 (21.1)	35 (28.2)	
**Work frequency past 6 months**				**0.016**
Daily	29 (36.7)	22 (17.4)	46 (29.7)	
Weekly	38 (48.1)	31 (40.8)	69 (44.5)	
Monthly or less	12 (15.2)	28 (36.8)	40 (25.8)	
**Reasons for self-employed sex work**				
Wanted to be independent	21 (26.6)	6 (7.9)	27 (17.4)	**0.002**
Bad experience with pimp, partner	8 (10.1)	2 (2.6)	10 (6.5)	0.058
For the money	48 (60.8)	31 (40.8)	79 (51.0)	**0.013**
Freedom in working hours	31 (39.2)	14 (18.4)	45 (29.0)	**0.004**
I like sex	27 (34.2)	39 (51.3)	66 (42.6)	**0.031**
Need money for drugs	9 (11.4)	1 (1.3)	10 (6.5)	**0.011**
Already had casual partners, made it my job	13 (16.5)	7 (9.2)	20 (12.9)	0.179
Because of a friend	10 (12.7)	7 (9.2)	17 (11.0)	0.492
Other	7 (8.9)	4 (5.3)	11 (7.1)	0.383
**Other job besides sex work**				**0.000**
Yes	27 (40.3)	41 (71.9)	68 (54.8)	
No	40 (59.7)	16 (28.1)	56 (45.2)	
**Main job**				0.084
Sex work	7 (25.9)	4 (10.0)	11 (16.4)	
Other job	20 (74.1)	36 (90.0)	56 (83.6)	
**Work location**				
Window	7 (8.9)	0 (0.0)	7 (4.5)	**0.008**
Private club	16 (20.3)	5 (6.6)	21 (13.5)	**0.013**
At home	28 (35.4)	28 (35.4)	56 (36.1)	0.856
Club	10 (12.7)	2 (2.6)	12 (7.7)	**0.020**
At client's home	24 (30.4)	35 (46.1)	59 (38.1)	**0.045**
Hotel room	27 (34.2)	30 (39.5)	57 (36.8)	0.494
Home of third party	11 (13.9)	12 (15.8)	23 (14.8)	0.744
Care institution	2 (2.5)	6 (7.9)	8 (5.2)	0.131
Swingers club	8 (10.1)	7 (9.2)	15 (9.7)	0.847
Massage parlour	7 (8.9)	2 (2.6)	9 (5.8)	0.097
Streets	7 (8.9)	1 (1.2)	8 (5.2)	**0.034**
Other	6 (7.6)	8 (10.5)	14 (9.0)	0.524
**STI risk behaviour**				
**Types of sex during work**				
Oral sex	65 (82.3)	68 (89.5)	133 (85.8)	0.199
Vaginal sex	68 (86.1)	60 (78.9)	128 (82.6)	0.242
Receptive anal sex	33 (41.8)	27 (35.5)	60 (38.7)	0.425
Insertive anal sex	n.a	43 (56.6)	n.a	n.a
BDSM or fetish	27 (34.2)	15 (19.7)	42 (27.1)	**0.043**
Use sex toys	41 (51.9)	42 (55.3)	83 (53.5)	0.675
Erotic massage with manual climax	52 (65.8)	44 (57.9)	96 (61.9)	0.310
Erotic massage with oral climax	44 (55.7)	48 (63.2)	92 (59.4)	0.344
**Inconsistent condom use during work**				
When giving oral sex	44 (62.0)	27 (62.8)	71 (62.3)	0.930
When receiving oral sex (only men)	n.a	56 (80.0)	n.a	n.a
With vaginal sex	35 (35.7)	21 (29.6)	46 (32.6)	0.437
With receptive anal sex	18 (46.2)	15 (35.7)	44 (40.7)	0.339
With insertive anal sex (only men)	n.a	14 (25.9)	n.a	n.a
**Unprotected sex at clients’ demand**				0.861
Yes	24 (32.9)	25 (34.2)	49 (33.6)	
No	49 (67.1)	48 (65.8)	97 (66.4)	
**Ask more money for unprotected sex**				**0.023**
Yes	21 (87.5)	14 (58.3)	35 (72.9)	
No	3 (12.5)	10 (41.7)	13 (27.1)	
**Ask more money for special requests**				**0.007**
Yes	42 (59.2)	26 (36.6)	68 (47.9)	
No	29 (40.8)	45 (63.4)	74 (52.1)	
**Alcohol use during work**				0.300
Yes	20 (25.3)	14 (18.7)	34 (21.9)	
No	59 (74.7)	62 (81.6)	121 (78.1)	
**Drug use during work**				0.103
Yes	27 (34.2)	17 (22.4)	44 (28.4)	
No	52 (65.8)	59 (77.6)	111 (71.6)	
**More often unprotected sex with alcohol or drugs**				0.708
Yes	13 (43.3)	8 (38.1)	21 (41.2)	
No	17 (56.7)	13 (61.9)	30 (58.8)	
**Group sex during work**				0.739
Yes	33 (45.2)	31 (42.5)	64 (43.8)	
No	40 (54.8)	42 (57.5)	82 (56.2)	
**STI testing**				
**STI testing past year (self-reported)**				0.946
Yes	47 (67.1)	44 (67.7)	91 (67.4)	
No	23 (32.9)	21 (32.3)	44 (32.6)	
**STI test location(s) of STI test(s) in past year**^b^				
STI clinic	25 (53.2)	19 (43.2)	44 (48.4)	0.359
General practitioner	18 (38.3)	21 (47.7)	39 (42.9)	0.487
Hospital	5 (10.6)	3 (6.8)	8 (8.8)	0.503
Home-test	2 (4.3)	6 (13.6)	8 (8.8)	0.131
Other	6 (12.8)	1 (2.2)	7 (7.7)	0.060
**Main reasons for last STI testing**				
Routine screening	33 (50.8)	34 (68.0)	67 (58.3)	0.710
Partner notification	5 (7.7)	3 (6.0)	8 (7.0)	0.777
Unprotected sex	13 (20.0)	6 (12.0)	19 (16.5)	0.104
STI related symptoms	3 (4.6)	1 (2.0)	4 (3.5)	0.330
Condom failure	7 (10.8)	4 (8.0)	11 (9.6)	0.383
Other	4 (6.2)	2 (4.0)	6 (5.2)	0.433
**Had an STI in the past year (self-reported)**				0.500
Yes	8 (11.6)	5 (8.1)	13 (9.9)	
No	61 (88.4)	57 (91.9)	118 (90.1)	

^a^Percentages may not precisely add up to 100% due to rounding

^b^Only answered if positive with regards to STI testing last year, more than one answer was possible

In bold, statistically significant (*p* < 0.05)

*n.a.* Not applicable, *BDSM* Bondage, Domination, Sadism and Masochism

Table 1 shows that compared with MSW, FSW more often had a country of birth other than the Netherlands (34.2% FSW vs. 19.7% MSW, *p* = 0.043). Most FSW and MSW had an intermediate or high educational level (17.7% FSW and 7.9% MSW had a low educational level, *p* = 0.154). Both FSW and MSW more often had sex with opposite sex partners in their private life (63.3% FSW and 64.5% MSW, *p* = 0.979) as well as during work (65.8% FSW and 61.6% MSW, *p* = 0.085). FSW more often worked in the sex industry for 1 to 5 years compared with MSW (49.3% FSW and 42.1% MSW, *p* = 0.027). MSW worked daily less frequently than FSW (36.7% FSW and 17.4% MSW) and they more often worked monthly or less than FSW (15.2% FSW and 36.8% MSW, *p* = 0.016). Compared with MSW, FSW reported more often that the main reason to engage in self-employed sex work was “for the money” (60.8% FSW and 40.8% MSW, *p* = 0.013). MSW reported “I like sex” more often to be the main reason for self-employed sex work than FSW (51.3% MSW and 34.2% FSW, *p* = 0.031). Compared with MSW, FSW worked more often in a window (8.9% FSW vs. 0.0% MSW, *p* = 0.008), at a private club (20.3% FSW vs. 6.6% MSW, *p* = 0.013) or at a club (12.7% FSW and 2.6% MSW, *p* = 0.020), while MSW worked more often at a client's home than FSW (30.4% FSW vs. 46.1% MSW, *p* = 0.045).

### STI risk behaviour and STI testing

During oral sex, 62.0% of FSW and 62.8% of MSW did not always use a condom (*p* = 0.930). When practicing vaginal sex, 35.7% of FSW and 29.6% of MSW did not always use a condom (*p* = 0.437). Furthermore, 46.2% of FSW and 35.7% of MSW did not always use a condom during receptive anal sex (*p* = 0.339), although these findings were not significantly different between FSW and MSW. When more money was offered, FSW consented to unsafe sex and other special requests more often than MSW (87.5% FSW vs. 58.3% MSW, *p* = 0.023, for unsafe sex and 59.2% FSW vs 36.6% MSW, *p* = 0.007, for special requests). Alcohol use during work was reported by 25.3% of FSW and 18.7% of MSW (*p* = 0.300), and drug use by 34.2% of FSW and 28.4% of MSW (*p* = 0.103).

Regarding STI testing, 67.1% of FSW and 67.7% of MSW underwent STI testing in the past year (*p* = 0.946). Most FSW went to an STI clinic for the test (53.2% FSW and 43.2% MSW, *p* = 0.359), while most MSW went to a general practitioner (47.7% MSW and 38.3% FSW, *p* = 0.487). Of all sex workers who participated in this survey, 67.5% were aware that the STI clinics in the Netherlands offered low threshold STI testing for sex workers (data not displayed in Table 1). The main reason for STI testing for both FSW and MSW was routine screening (58.0% FSW and 68.0% MSW, *p* = 0.710). In the past year, 11.6% of FSW and 8.1% of MSW reported having an STI (*p* = 0.500).

### Reported STI testing in the past year

Table 2 shows factors for self-reported STI testing over the past year. Only “work years” was significantly associated with STI testing in the past year, although this was not a linear association. Other determinants were not significantly associated with undergoing an STI test in the past year.

**Table 2:** Factors of self-reported STI testing in the past year among self-employed female and male sex workers in the Netherlands (2019–2020)

	**STI testing past year** n (%)^a^	**No STI testing past year** n (%)^a^	***p*-value**
**Demographics**			
**Gender**			0.946
Female	47 (67.1)	23 (32.9)	
Male	44 (67.7)	21 (32.3)	
**Country of birth**			0.747
The Netherlands	68 (66.7)	34 (33.3)	
Other country	23 (69.7)	10 (30.3)	
**Educational level**			0.542
Low educational level	12 (66.7)	6 (33.3)	
Intermediate educational level	46 (71.9)	18 (28.1)	
High educational level	33 (62.3)	20 (37.7)	
**Relationship status**			0.439
Relationship	33 (63.5)	19 (36.5)	
Single	58 (69.9)	25 (30.1)	
**Gender private sex partners**			0.335
Same sex	7 (70.0)	3 (30.0)	
Opposite sex	62 (71.3)	25 (28.7)	
Both opposite and same sex	22 (57.9)	16 (42.1)	
**Gender work sex partners**			0.538
Same sex	9 (81.8)	2 (18.2)	
Opposite sex	57 (67.1)	28 (32.9)	
Both opposite and same sex	25 (64.1)	14 (35.9)	
**Sex work**			
**Work years**			**0.021**
< 1 year	20 (62.5)	12 (37.5)	
1–5 year	46 (80.7)	11 (19.3)	
> 5 year	19 (54.3)	16 (45.7)	
**Work frequency past 6 months**			0.640
Daily	30 (73.2)	11 (26.8)	
Weekly	39 (65.0)	21 (35.0)	
Monthly or less	22 (64.7)	12 (35.3)	
**STI risk behaviour**			
**Inconsistent condom use during work**			
*When giving oral sex*			0.942
Yes	45 (68.2)	21 (31.8)	
No	27 (67.5)	13 (32.5)	
*When receiving oral sex (only men)*			0.172
Yes	36 (73.5)	13 (26.5)	
No	7 (53.8)	6 (46.2)	
*With vaginal sex*			0.168
Yes	26 (60.5)	17 (39.5)	
No	63 (72.4)	24 (27.6)	
*With receptive anal sex*			0.284
Yes	19 (61.3)	12 (38.7)	
No	30 (73.2)	11 (26.8)	
*With insertive anal sex (only men)*			0.581
Yes	9 (81.8)	2 (18.2)	
No	28 (73.7)	10 (26.3)	
**Alcohol use during work**			0.417
Yes	21 (61.8)	13 (38.2)	
No	70 (69.3)	31 (30.7)	
**Drug use during work**			0.516
Yes	28 (63.6)	16 (36.4)	
No	63 (69.2)	28 (30.8)	
**Group sex during work**			0.680
Yes	40 (65.6)	21 (34.4)	
No	51 (68.9)	23 (31.1)	

^a^Percentages may not precisely add up to 100% due to rounding

In bold, statistically significant (*p* < 0.05)

## Discussion

Our study shows that self-employed sex workers have a heterosexual preference, both at work and in private life (approximately two-thirds of FSW and MSW). Approximately two-thirds of FSW and MSW did not always use a condom while giving oral sex and one-third with vaginal sex, and half of FSW, and one-third of MSW did not always use a condom during receptive anal sex.

Not testing for STI in the past year was still substantial with one-third of both FSW and MSW and one-third of all participating sex workers being unaware of the possibility getting a low-threshold test at an STI clinic. Although condom use and STI testing are not consistent among all sex workers, the self-reported STI positivity is relatively low (around 10%).

### Strengths and limitations

Our study provides more insight into the group of self-employed sex workers, which is of value in addition to the existing literature on sex workers when self-employment is mostly unknown.

However, this study has several limitations. Overall, the response to our survey was low, despite relatively intense efforts to recruit participants. Although we clearly stated that the participation in this study was fully anonymous, we know from interviews that trust is an issue with sex workers, and therefore, many would likely not participate in a survey because of anticipated governmental consequences.

Furthermore, the stigmatized subject of the survey might have led to socially desirable responses. Moreover, the survey relied on self-reporting, which could have led to a recall bias. This recall bias and socially desirable responses might have yielded lower outcomes for certain variables, such as having had an STI in the past year.

Of those who participated, the majority had a higher educational level, suggesting selection-bias. This might result in an underestimation of risk, as lower-educated sex workers tend to use even fewer condoms during work and have fewer STI consultations [15]. Due to advertising mainly on heterosexual sex advertising websites, a selection bias occurred, resulting in an under-representation of MSW who have sex with men and TSWs.

### Comparison to other studies

Our study showed that inconsistent condom use for both FSW and MSW ranged from 33% during vaginal sex to 62% during oral sex (i.e., consistent condom use ranged from 67% during vaginal sex and 38% during oral sex). Our findings are in line with an Australian study reporting approximately the same percentages of consistent condom use during sex (33% during oral sex, 67% during vaginal sex, and 59% during anal sex) among female, male and transgender sex workers [18]. Other studies have shown higher rates of consistent condom use among Dutch sex workers (around 80%) [4, 19]. However, these studies focused on sex workers in general, whereas we specifically addressed self-employed sex workers, which suggests they tend to be more vulnerable to STIs. Furthermore, two studies showed that condom use is influenced by work environments; female sex workers working exclusively in brothels reported higher rates of condom use compared with those working privately, however, the self-employment of sex workers was not taken into account [18, 19].

Regarding STI testing, our study showed that two-thirds of both FSW and MSW had reported performing an STI test in the past year. This finding is in line with other study findings, which also reported STI test rates ranging between 56 and 86% [19, 20]. Similar to condom use, testing for STIs is also influenced by the work environment and one study found that sex workers working in the streets had lower testing rates (56%), than those who worked in multiple settings (streets, venues, and online, 86%) [20].

For FSW, the findings regarding STI positivity were in line with other studies [14, 15, 21]. However, STI positivity rates among MSW were higher in other studies compared with our results [9, 14, 22]. These studies had higher numbers of MSW who had sex with men compared with our study and did not use self-report in a questionnaire, but used laboratory-confirmed STI diagnoses, which might explain the difference in STI positivity rates.

### Interpretations

Although a lot of effort was put into reaching self-employed sex workers, many did not participate in the study. Often, there is distrust of public authorities, such as the STI clinic of the public health service who initiated the study [2]. This is due to the fact that many cities in the Netherlands have legislations against working from home, with a risk of being fined or forced to come out as a sex worker when caught, sometimes even leading to eviction of the sex worker from his or her home [2]. Moreover, self-employed sex workers need permits to practice their profession. With the application for a permit, the address of the sex worker is placed on the municipal website, which threatens the safety of the self-employed sex worker [2]. This legislation forces sex workers to work in illegal circuits and isolated work locations, disrupting peer support networks and service access and limiting risk reduction opportunities [11]. In addition to limited risk reduction opportunities, increasing client demand for condomless sex and concerns about income reductions if condomless sex is not offered appear to be factors related to the provision of condomless sex [18].

In contrast, what strengthens their position is that self-employed sex workers have a similar position on the labour market than other non-sex-related self-employed workers. They determine their own terms and conditions, choose their own work settings and times and set their own rates [23].

There are various outreach initiatives in the Netherlands to reach sex workers, such as offering STI testing at their work location and Internet fieldwork on websites where sex workers advertise. Considering our study findings that one-third of self-employed sex workers did not undergo an STI test in the past year and that one-third of the participants were unaware of the possibility of getting low-threshold STI testing at an STI clinic, these outreach activities might not be enough to bridge the testing gap.

Although our study provides a starting point for gathering more knowledge on self-employed sex workers, much is still unknown. Because our study suffered from a selection bias, more research is needed on the STI risk behaviour and test behaviour of self-employed sex workers with a lower educational level and non-Dutch self-employed sex workers. Moreover, self-employed MSW who have sex with men during work and TSW were underrepresented in our study, which warrants further research on these groups.

## Conclusion

In conclusion, our study shows that approximately two-thirds of FSW and MSW had performed an STI test in the past year, and the STI positivity rate for this population was not very high. However, tailored STI prevention strategies remain important considering the low compliance with condom use during work and the large number of sexual contacts that might facilitate spread when STI positive. As self-employed sex workers appear to be a hidden population, this continues to be a challenge with regard to future policy and research for professionals in the field of sexual healthcare.

## Data Availability

The datasets used and/or analysed during the current study are available from the corresponding author on reasonable request.

## Abbreviations

CI: Confidence Interval
FSW: Female (self-employed) sex worker
HIV: Human Immunodeficiency Virus
MSW: Male (self-employed) sex worker
STI: Sexually Transmitted Infections
TSW: Transgender (self-employed) sex worker
